# Editorial: Education in ethnopharmacology 2022

**DOI:** 10.3389/fphar.2023.1154280

**Published:** 2023-02-10

**Authors:** Oliver Grundmann, Devina Lobine, James Olukayode Olopade

**Affiliations:** ^1^ College of Pharmacy, University of Florida, Gainesville, FL, United States; ^2^ Faculty of Life Science, JSS Academy of Higher Education and Research, Bonne Terre, Mauritius; ^3^ Faculty of Veterinary Medicine, University of Ibadan, Ibadan, Nigeria

**Keywords:** ethnopharmacology, education, traditional medicine, pharmacy, healthcare

Healing with medicinal plants is as old as mankind itself. Their medicinal uses in thousands of reports evidences that plants have been serving as a mainstay for pharmacotherapy up until the last century. Ethnopharmacology is an interdisciplinary exploration of active principles in historical and traditional uses of plants and other sources for medical therapy and thus, incorporates concepts and methods from ethnobotany, medical anthropology, and pharmacognosy, among others.

The current landscape of ethnopharmacology has centered on traditional plant medicines that are often part of an established medical system, such as Ayurveda, Traditional Chinese Medicine (TCM), or Shamanism. Knowledge about traditional medicinal remains sparse outside of the ethnic group using the medicine and is often passed on by oral tradition. In many instances, the pharmacologically active ingredients and their mechanisms of action are unknown although there is substantial interest to identify active principles for developing pharmaceuticals and lead substances in drug development. Ethnopharmacological research remains prevalent in several countries with a rich history of traditional medicines such as China, India, South America, Africa, and parts of Europe. However, few institutions in the US and other countries offer formal training or education in ethnopharmacology and focus primarily on synthetic pharmaceutical substances used in modern pharmacotherapy. This limits education in this field to maintain research, appropriately train skilled professionals, and provide guidance to patients who wish to take traditional medicines.

One such plant with a rich history in TCM is *Angelica sinensis (Oliv.) Diels*, also known as ginseng or Danggui in Chinese. Because of its widespread use, much of its research over recent years has been conducted by Chinese investigators followed by Taiwan, South Korea, the United States, and Japan (Lu et al.). Research on *Angelica sinensis* has primarily focused on its biological activities and the main pharmacologically active ingredients, ferulic acid and butylidenephthalide.

Diversity of medicinal plants is of particular interest given a changing climate and socioeconomic factors contributing to a sustainable ecosystem. A study tracked medicinal plant diversity in Mongolia over 9 years to determine which particular factors impact plant diversity. The research determined that temperature and precipitation fluctuations as well as socioeconomic development, including an interest in particular medicinal plants for commercial purposes, correlate with medicinal plant diversity in inner Mongolia (Zhang et al.).

In recent years, the use of herbal supplements has steadily increased, in part attributable to the ongoing COVID-19 pandemic. Pharmacists serve an essential public health role in evaluating medications for individual patients and the respective quality. Given the multitude of herbal supplements available, a tool was created and validated based on five factors to allow pharmacists evaluation of supplements based on label statements for intended use, container and hazard identification, product authenticity indicator, packaging and product insert, and handling and storage measures (Jairoun et al.). The tool achieved a high content validity index of 0.891 with a Cronbach’s alpha of 0.940 indicating good fit. This tool may hence serve pharmacists to better identify quality herbal supplements and counsel patients on their use.

In a formal education setting, many institutions have adopted a flipped classroom approach with lectures being recorded and viewed prior to attending a practice or active or learning session where learners apply what they learned during lecture. Comparison between a flipped classroom approach and traditional lecture style in Chinese pharmacy students indicate that the former leads to significantly better test and learning measure outcomes compared to traditional lectures (Peng et al.). The use of flipped classroom designs may thus improve pharmacy education, including knowledge and skill development in traditional medicines and ethnopharmacology.

We hope that this Research Topic provides readers with insights into the diverse educational efforts in ethnopharmacology. While some of the studies directly relate to formal education and the practice of traditional medicine, others address more complex Research Topic impacting plant diversity and knowledge about their pharmacology.

